# The 14-3-3 protein CaTFT7 interacts with transcription factor CaHDZ27 to positively regulate pepper immunity against *Ralstonia solanacearum*

**DOI:** 10.1093/hr/uhaf010

**Published:** 2025-01-14

**Authors:** Shaoliang Mou, Xiaodan Chen, Jiao Cai, Tingting Zhang, Tong Luo, Shuilin He

**Affiliations:** College of Life Science, Fujian Agriculture and Forestry University, No.15 Shangxiadian Road, Cangshan District, Fuzhou City, 350002, Fujian Province, China; Key Laboratory of Applied Genetics of Universities in Fujian Province, Fujian Agriculture and Forestry University, No.15 Shangxiadian Road, Cangshan District, Fuzhou City, 350002, Fujian Province, China; National Education Ministry Key Laboratory of Plant Genetic Improvement and Comprehensive Utilization Fujian Agriculture and Forestry University, No.15 Shangxiadian Road, Cangshan District, Fuzhou City, 350002, Fujian Province, China; College of Life Science, Fujian Agriculture and Forestry University, No.15 Shangxiadian Road, Cangshan District, Fuzhou City, 350002, Fujian Province, China; Key Laboratory of Applied Genetics of Universities in Fujian Province, Fujian Agriculture and Forestry University, No.15 Shangxiadian Road, Cangshan District, Fuzhou City, 350002, Fujian Province, China; National Education Ministry Key Laboratory of Plant Genetic Improvement and Comprehensive Utilization Fujian Agriculture and Forestry University, No.15 Shangxiadian Road, Cangshan District, Fuzhou City, 350002, Fujian Province, China; College of Life Science, Fujian Agriculture and Forestry University, No.15 Shangxiadian Road, Cangshan District, Fuzhou City, 350002, Fujian Province, China; Key Laboratory of Applied Genetics of Universities in Fujian Province, Fujian Agriculture and Forestry University, No.15 Shangxiadian Road, Cangshan District, Fuzhou City, 350002, Fujian Province, China; National Education Ministry Key Laboratory of Plant Genetic Improvement and Comprehensive Utilization Fujian Agriculture and Forestry University, No.15 Shangxiadian Road, Cangshan District, Fuzhou City, 350002, Fujian Province, China; College of Life Science, Fujian Agriculture and Forestry University, No.15 Shangxiadian Road, Cangshan District, Fuzhou City, 350002, Fujian Province, China; Key Laboratory of Applied Genetics of Universities in Fujian Province, Fujian Agriculture and Forestry University, No.15 Shangxiadian Road, Cangshan District, Fuzhou City, 350002, Fujian Province, China; National Education Ministry Key Laboratory of Plant Genetic Improvement and Comprehensive Utilization Fujian Agriculture and Forestry University, No.15 Shangxiadian Road, Cangshan District, Fuzhou City, 350002, Fujian Province, China; College of Life Science, Fujian Agriculture and Forestry University, No.15 Shangxiadian Road, Cangshan District, Fuzhou City, 350002, Fujian Province, China; Key Laboratory of Applied Genetics of Universities in Fujian Province, Fujian Agriculture and Forestry University, No.15 Shangxiadian Road, Cangshan District, Fuzhou City, 350002, Fujian Province, China; National Education Ministry Key Laboratory of Plant Genetic Improvement and Comprehensive Utilization Fujian Agriculture and Forestry University, No.15 Shangxiadian Road, Cangshan District, Fuzhou City, 350002, Fujian Province, China; Key Laboratory of Applied Genetics of Universities in Fujian Province, Fujian Agriculture and Forestry University, No.15 Shangxiadian Road, Cangshan District, Fuzhou City, 350002, Fujian Province, China; National Education Ministry Key Laboratory of Plant Genetic Improvement and Comprehensive Utilization Fujian Agriculture and Forestry University, No.15 Shangxiadian Road, Cangshan District, Fuzhou City, 350002, Fujian Province, China; College of Agriculture Science, Fujian Agriculture and Forestry University, No.15 Shangxiadian Road, Cangshan District, Fuzhou City, 350002, Fujian Province, China

## Abstract

Bacterial wilt, caused by *Ralstonia solanacearum*, is a devastating disease affecting plants in the Solanaceae family. In our previous study, CaHDZ27 was shown to act crucially in the pepper defense response to *R. solanacearum*. However, the molecular basis underlying CaHDZ27 function remains unexplored. In this study, we demonstrate that CaHDZ27 is post-translationally regulated by the 14-3-3 protein CaTFT7, which functions as a positive regulator in pepper immunity against *R. solanacearum*. RT-qPCR analysis revealed that CaTFT7 is transcriptionally induced by *R. solanacearum* infection. The data from virus-induced gene silencing revealed that *CaTFT7* positively affects pepper immunity, which was further confirmed by the data of CaTFT7-overexpressing *Nicotiana benthamiana*. CaTFT7 interacted with CaHDZ27, thereby promoting the stability of CaHDZ27 and enhancing CaHDZ27 binding to the promoter of *cysteine-rich receptor-like protein kinase 5* (*CaCRK5*), a gene that positively affects pepper defense against *R. solanacearum*. The above data indicated that CaTFT7 enhanced CaHDZ27 stability and promoted its ability to activate pepper immunity, shedding light on the mechanisms underlying pepper resistance to bacterial wilt.

## Introduction

Plants face constant challenges from pathogenic microbes throughout their growth and development. Over the course of evolution, plants have developed two major strategies to detect and combat pathogens: pattern-triggered immunity (PTI) and effector-triggered immunity (ETI) [1, 2]. These complex immune responses depend on the activation of a cascade of molecular networks, which are usually accompanied by a large amount of transcriptional reprogramming. Transcription factors (TFs) play vital roles in this process [3, 4]; however, the mechanisms ensuring their stability and proper functioning are not fully understood.

HD-Zip proteins are a family of plant-specific TFs characterized by a conserved homeodomain and an adjacent leucine zipper domain [5–7]. The homeodomain binds specific DNA sequences, while the leucine zipper facilitates protein–protein interactions [8]. Based on their DNA-binding motifs, additional conserved motifs, and gene structures, HD-Zip proteins has been divided into four subfamilies [5]. HD-Zip I proteins recognize the specific HDZ motif CAAT(A/T)ATTG and tend to play roles in plant tolerance to many abiotic stresses, such as drought and salt [9, 10]. Recent studies have revealed that several HD-Zip I proteins participate in plant immune responses. For example, the overexpression of *ATHB13* enhanced Arabidopsis resistance to downy mildew and green peach aphid [11]. MdHB-7 plays a negative role in apple immunity against *Colletotrichum fructicola* [12]. In our previous study, CaHDZ27 positively regulated pepper defense responses against *Ralstonia solanacearum* [13]; CaHDZ27 directly targeted the disease resistance-related genes *CaCRK5* and *CaLRR-RLK1* and boosted their transcript levels [14, 15]. Despite these findings, the role of HD-Zip I proteins in mediating plant immune responses remains poorly understood.

The 14-3-3 proteins are a conserved family of regulatory proteins found in all eukaryotes. Originally discovered in the mammalian brain, they were named for their migration patterns on starch gel electrophoresis [16]. These proteins can interact with many kinds of proteins in a phosphorylation-dependent manner [17], thereby mediating distinct physiological processes. In plants, there are many 14-3-3 proteins, e.g. 12 in tomato and 13 in *Arabidopsis* [18]. The 14-3-3 proteins are key factors regulating plant disease resistance. In rice, the 14-3-3 protein GF14b mediates disease resistance to leaf blast via the jasmonic acid and salicylic acid signaling pathways [19]. In *Arabidopsis*, GF14λ positively regulates resistance protein RPW8-mediated resistance to powdery mildew [20]. 14-3-3 proteins exert their functions by affecting subcellular localization, stability, or activities of their binding partners [21, 22]. An increasing number of studies indicate that 14-3-3 proteins regulate the functions of TFs, thereby mediating biotic and abiotic responses. For example, rice GF14c positively regulates disease resistance by affecting homoeostasis of OsSCL7 [23]. Apple MdGRF8 modulates salt stress tolerance by increasing transcriptional activity and stability of MdWRKY18 [24]. However, the molecular mechanisms by which 14-3-3 proteins collaborate with TFs to regulate plant immunity remain unclear.

**Figure 1:**
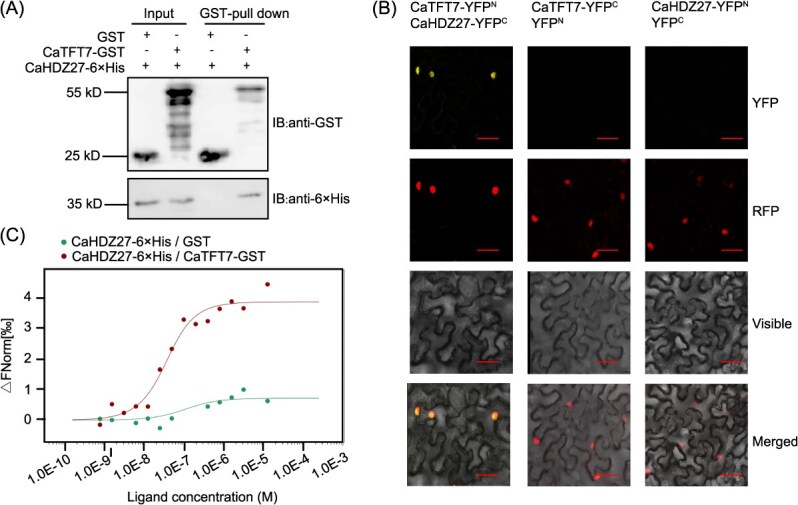
CaTFT7 physically interacts with CaHDZ27, which contributes to pepper resistance against *R. solanacearum*. (A) Pull-down analysis revealed that CaTFT7-GST interacted with CaHDZ27–6 × His. Purified CaTFT7-GST or GST was incubated with CaHDZ27–6 × His, and then pulled down through the GST beads. The proteins were determined with anti-His and anti-GST antibodies, respectively. (B) BiFC assays revealed that CaTFT7 interacted with CaHDZ27 in *N. benthamiana* leaf epidermal cells. NbH2B (histone H2B)-RFP indicated the nucleus. Bars = 50 μm. (C) MST assay determined the interaction between CaTFT7 and CaHDZ27. CaTFT7-GST was the target, and CaHDZ27–6 × His was regarded as the ligand.

*Ralstonia solanacearum*, the causal agent of bacterial wilt, infects Solanaceae species such as tomato, pepper, and eggplant. This soil-borne bacterial pathogen enters hosts through root wounds, multiplies in the xylem vessels, and produces exopolysaccharides, ultimately obstructing the water transport system and resulting in plant mortality [15, 25–28]. In this study, we reveal that the 14-3-3 protein CaTFT7 enhances pepper resistance to *R. solanacearum*. *CaTFT7*-silenced pepper plants present compromised resistance to wilt disease, whereas *CaTFT7*-overexpressing *Nicotiana benthamiana* plants exhibit increased resistance. Furthermore, we found that CaTFT7 interacts with the HD-Zip I protein CaHDZ27, stabilizing it and promoting its transcriptional activity. These findings provide new insights into the role of 14-3-3 proteins in plant immunity and their interaction with TFs to combat bacterial wilt.

## Results

### Identification of CaTFT7 interacting with CaHDZ27

In our previous study, the HD-Zip I protein CaHDZ27 was proved to contribute to pepper resistance to wilt disease [13]. To investigate the regulatory mechanisms by which CaHDZ27 contributes to pepper immunity, we performed a glutathione *S*-transferase (GST) pull-down assay combined with mass spectrometry to screen proteins of interacting with CaHDZ27. Among the 10 proteins identified (Table S1), a 14-3-3 protein (CaTFT7, XP_016569248.1) attracted our interest since 14-3-3 proteins are known to regulate TFs and play pivotal roles in plant disease resistance. The full-length cDNA of the 14-3-3 gene was cloned, encoding a predicted protein of 251 amino acids. Phylogenetic analysis of CaTFT7 and 14-3-3 family proteins obtained from the *Arabidopsis* and tomato genome databases revealed that our 14-3-3 protein and SlTFT7 are closely related, so it was designed as CaTFT7 (Fig. S1a). Sequence alignment and domain analysis indicated that CaTFT7 and SlTFT7 presented rather high sequence similarity (99.21%), with a very conserved 14-3-3 domain (Fig. S1b). Tomato SlTFT7 was reported to positively regulate immunity-related programmed cell death, which was mediated by both Pto and MAPKKKα (mitogen-activated protein kinase kinase) [29, 30], suggesting that CaTFT7 may play a similar role in pepper immunity.

To validate the physical interaction between CaTFT7 and CaHDZ27, pull-down assays were first performed. CaTFT7 and CaHDZ27 were fused with GST and 6 × His tags, respectively. CaHDZ27 was detected by anti-His antibodies via western blotting of proteins eluted from CaTFT7-GST beads, whereas no CaHDZ27 was detected from free GST beads (Fig. 1a). The interaction was subsequently validated through bimolecular fluorescence complementation (BiFC) assay. CaTFT7 was fused with the N-terminal fragment of YFP (CaTFT7-YFP^N^), while CaHDZ27 was fused with the C-terminal fragment of YFP (CaHDZ27-YFP^C^). Transient coexpression of CaTFT7-YFP^N^ and CaHDZ27-YFP^C^ in *N. benthamiana* leaves was performed, and strong YFP fluorescence signals in the nucleus were observed from the interaction between CaTFT7 and CaHDZ27 at 48 h postinfiltration (hpi). No fluorescence signals were observed in the negative controls (Fig. 1b). In addition, the CaTFT7-CaHDZ27 interaction was confirmed via microscale thermophoresis (MST) analysis (Fig. 1c). Collectively, these results demonstrated that CaTFT7 directly interacted with CaHDZ27 in the nucleus.

### *CaTFT7* gene expression pattern and protein subcellular localization

**Figure 2:**
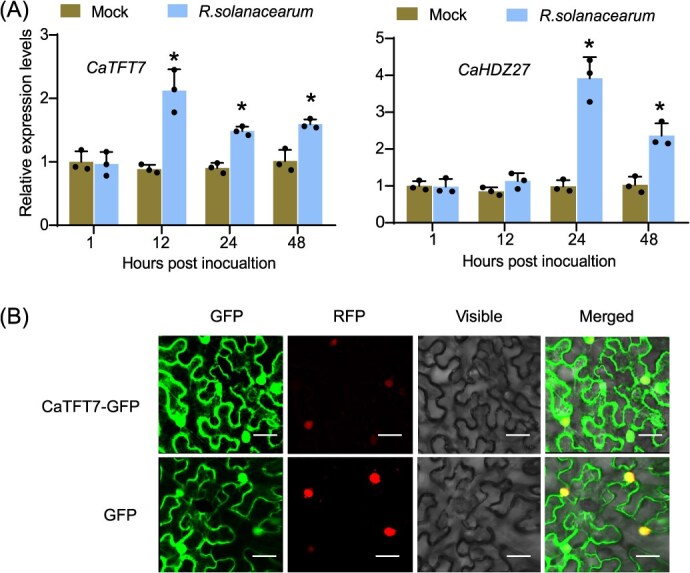
Expression analysis and subcellular localization of CaTFT7. (A) *CaTFT7* and *CaHDZ27* expression in 5-week-old pepper leaves challenged with *R. solanacearum* by RT-qPCR. The values are the means ± SDs from three independent biological replicates. Asterisks represent significant differences (^*^P <  0.05). (B) Subcellular localization of CaTFT7. The fusion construct (CaTFT7-GFP) and control (GFP) were transformed into *N. benthamiana* leaf epidermal cells; NbH2B (histone H2B)-RFP (red fluorescent protein) was the nuclear marker. Bar = 50 μm.

Our previous study revealed that CaHDZ27 plays a positive role in the defense response to *R. solanacearum* infection [13]. Since CaTFT7 physically interacted with CaHDZ27, we hypothesized that CaTFT7 may also participate in the pepper defense response. To explore the potential roles of *CaTFT7* in pepper immunity, we analyzed its transcript levels in response to *R. solanacearum* infection using reverse transcription quantitative real-time PCR (RT-qPCR). Similar to *CaHDZ27*, the transcript level of *CaTFT7* increased in pepper plants upon the inoculation of *R. solanacearum*, and peaked at 12 hpi, suggesting that *CaTFT7* may also contribute to the pepper resistance against *R. solanacearum* (Fig. 2a)*.*

Since protein functionality is closely tied to its subcellular localization, we further investigated the localization of CaTFT7 *in planta* via transient expression in *N. benthamiana* plant. The results revealed that significant fluorescence was observed in both cytoplasm and nucleus when the *35S*:*CaTFT7-GFP* (green fluorescent protein) construct was transformed into *N. benthamiana* leaves at 48 hpi (Fig. 2b), indicating that CaTFT7 is localized to both cytoplasm and nucleus. This finding is consistent with some previous studies on 14-3-3 proteins including SlTFT7 [31].

### Silencing of *CaTFT7* enhances pepper susceptibility to *R. solanacearum*

**Figure 3:**
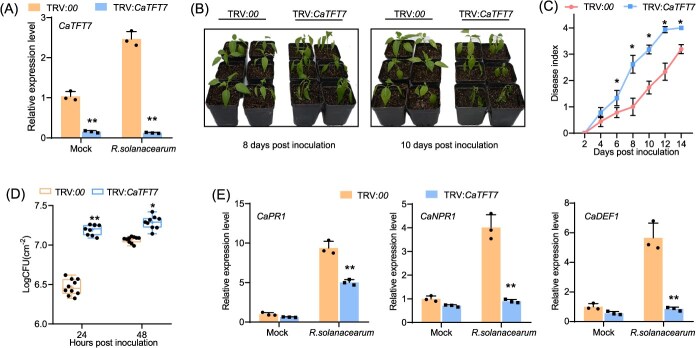
Increased susceptibility of *CaTFT7*-silenced pepper plants to *R. solanacearum* infection. (A) RT–qPCR analysis of *CaTFT7* silencing in pepper. Error bars represent ±SD of three biological replicates. ^**^P <  0.01 (Student’s *t-*test). (B) Phenotypic comparison of TRV:*CaTFT7* and TRV:*00* pepper plants root-inoculated with *R. solanacearum*. Five-week-old plants were challenged with *R. solanacearum* and grown at 28°C. Representative images of the infected plants were taken at 8 and 10 dpi. (C) Wilt disease progression was determined by rating plants on a scale from 0 to 4. The plotted values correspond to the means ± standard errors from three independent inoculations (*n* = 30 per line). ^*^P <  0.05 (Student’s *t*-test). (D) Growth of *R. solanacearum* in pepper leaves. Fifty microliters (10^7^ CFU ml^−1^) of bacterial suspension was infiltrated into 5-week-old TRV:*CaTFT7* and TRV:*00* pepper leaves. Bacterial growth was determined at 24 and 48 hpi (mean + SD, *n* = 8). ^*^P <0.05, ^**^P <  0.01 (Student’s *t*-test). (E) Expression of pathogenesis-related genes was determined via RT–qPCR. The values are presented as the means ± SDs of three independent experiments. ^**^P < 0.01 (Student’s *t-*test).

To gain more insights into the role of *CaTFT7* in pepper immunity, *CaTFT7* was silenced in pepper by virus-induced gene silencing (VIGS) assay. The 265-bp specific fragment of *CaTFT7* was selected for the construction of TRV:*CaTFT7.* When *TRV*:*CaPDS* pepper plants showed a bleaching phenotype (Fig. S2), the leaves were inoculated with *R. solanacearum.* At 12 hpi, the *CaTFT7* transcript level was checked via qRT–PCR analysis. The results showed a significant reduction in *CaTFT7* transcript abundance in TRV:*CaTFT7* plants compared to the TRV:*00* control plants (Fig. 3a).

Five-week-old TRV:*CaTFT7* and TRV:*00* control pepper plants were inoculated with *R. solanacearum* strain FJC100301 via soil-soak inoculation*.* An examination of the wilting phenotype revealed that TRV*:CaTFT7* plants showed more severe disease symptoms compared to TRV:*00* plants (Fig. 3b). The disease indices were distinctly greater in TRV*:CaTFT7* plants than in TRV:*00* plants (Fig. 3c). Furthermore, bacterial quantification revealed greater *R. solanacearum* density in the leaves of TRV:*CaTFT7* plants at 24 and 48 hpi, compared to the control plants (Fig. 3d).

The expression of immune marker genes (*CaPR1*, *CaNPR1*, and *CaDEF1*) was also evaluated via RT-qPCR. Upon *R. solanacearum* challenge, these genes showed reduced expression in TRV:*CaTFT7* plants compared to the TRV:*00* controls (Fig. 3e). In contrast, the expression of *CaHDZ27* was not significantly different between TRV:*CaTFT7* and TRV:*00* plants (Fig. S3), indicating that CaTFT7 does not regulate CaHDZ27 at the transcript level. On the basis of these data, we believe that *CaTFT7* participates in pepper defense responding to *R. solanacearum* infection.

### Overexpression of *CaTFT7* in transgenic *N. benthamiana* plants increased resistance to *R. solanacearum* infection

**Figure 4:**
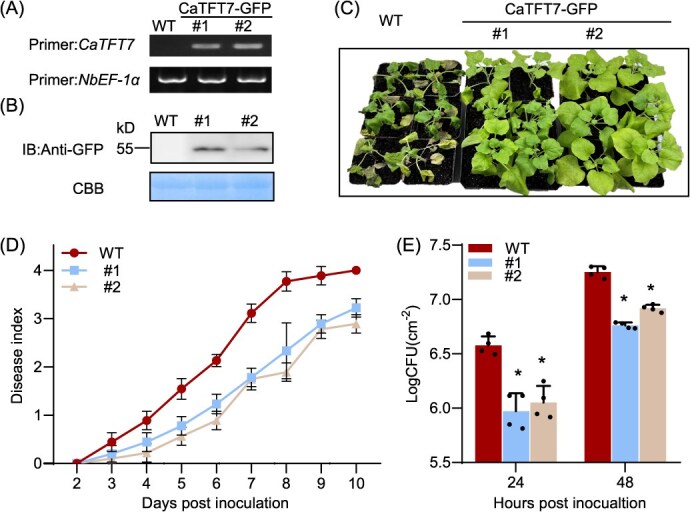
Enhanced resistance against *R. solanacearum* in *CaTFT7*-overexpressing transgenic *N. benthamiana* plants. (A) Expression of *CaTFT7* in transgenic and WT plants via RT-PCR. (B) Immunoblotting analysis of CaTFT7-GFP with an anti-GFP antibody. (C) Phenotypes of *CaTFT7*-overexpressing and WT plants challenged by *R. solanacearum*. Four-week-old plants were root-inoculated with 10^8^ CFU ml^−1^  *R. solanacearum* and then grown at 26°C. Representative images of wilting symptoms were taken at 7 dpi. (D) Disease indices of *CaTFT7*-overexpressing and WT plants inoculated with *R. solanacearum* from 0 to 10 days. The plotted values correspond to the means ± SDs from three independent inoculations (*n* = 30 per line). (E) Bacterial accumulation assays in *N. benthamiana* leaves at 24 and 48 hpi. The values are represented as the means ± SDs (*n* = 4). Asterisks indicate significant differences (*P* < 0.05) according to Student’s *t*-test.

To further clarify the biological function of *CaTFT7*, *N. benthamiana* plants ectopically overexpressing *CaTFT7* were generated in a stable fashion via *Agrobacterium-*mediated transformation. At least 10 T_3_ transgenic lines were obtained, two lines (#1 and #2) with expression of *CaTFT7* confirmed via RT–PCR (Fig. 4a) and western blotting by anti-GFP antibody (Fig. 4b), were chosen for disease resistance analyses.

Four-week-old wild-type (WT) and *CaTFT7*-overexpressing *N. benthamiana* plants were inoculated with *R. solanacearum*. We observed that the *CaTFT7*-overexpressing plants presented an enhanced resistant phenotype at 7 days postinoculation (dpi) (Fig. 4c). To further quantify the extent of disease, the disease index values were determined in the *CaTFT7*-overexpressing and WT plants from 2 to 10 dpi, and lower disease index values were presented in *CaTFT7*-overexpressing plants (Fig. 4d). Bacterial accumulation assays confirmed reduced *R. solanacearum* density in the leaves of transgenic plants at 24 and 48 hpi (Fig. 4e). These results demonstrate that overexpression of *CaTFT7* enhanced the resistance to *R. solanacearum.* Similar to *CaHDZ27*, *CaTFT7* also positively regulates pepper immunity.

### CaTFT7 enhances transcriptional activity and promotes the stability of transcription factor CaHDZ27

**Figure 5:**
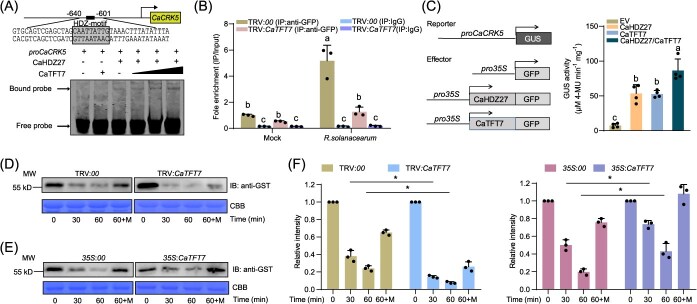
CaTFT7 regulated CaHDZ27 by increasing its transcriptional activity and increasing its stability. (A) EMSA was used to determine the effect of CaTFT7 on CaHDZ27 binding to the *CaCRK5* promoter. Cy5-labeled DNA probes were incubated with CaHDZ27-GST and/or CaTFT7-GST. (B) ChIP–qPCR analysis for CaHDZ27 binding to the *CaCRK5* promoter when CaTFT7 was silenced. 35S:CaHDZ27-GFP was transiently expressed in 5-week-old TRV:*CaTFT7* and TRV:*00* pepper plants. At 48 hpi, the leaves were harvested for crosslinking, followed by immunoprecipitation with anti-GFP. Different letters indicate significant differences by LSD test (*P* < 0.05). (C) Effect of CaTFT7 on CaHDZ27 transcriptional regulation of *CaCRK5. 35S*:*GFP* (empty vector), *35S*:*CaHDZ27-GFP*, and/or *35S*:*CaTFT7-GFP* were transiently expressed in *proCaCRK5*:*GUS* transgenic *N. benthamiana* plants. After 48 hpi, the GUS reporter activities of the *CaCRK5* promoter were measured. (D) Effects of CaTFT7 on CaHDZ27 protein stability. In cell-free degradation assay, recombinant purified CaHDZ27-GST was mixed with total protein extracted from TRV:CaTFT7 and TRV:00 pepper plants. CaHDZ27-GST was detected at the indicated time points via an anti-GST antibody. The equivalent loading of the crude extracts was determined by Coomassie Brilliant Blue (CBB) staining. M, MG132. (E) The cell-free degradation assay using *CaTFT7*-overexpressing *N. benthamiana* plants. (F) The abundance of CaHDZ27-GST in (D) and (E) was quantified via ImageJ software. The relative protein levels of CaHDZ27-GST at different time points were normalized to the value at 0 min (set as 1). The data are presented as the means ± SDs from three independent experiments. Asterisks represent significant differences (Student’s *t-*test; *P* < 0.05).

Previous studies established that 14-3-3 proteins can directly affect TFs by regulating their stability, subcellular localization, and transcriptional activity [23, 24, 32, 33]. *Cysteine-rich receptor-like kinase* (*CRK*) genes, which play important roles in the immune responses, are transcriptionally induced by pathogen attack [34, 35]. Our previous study revealed that the expression of *CaCRK5* was activated by CaHDZ27 binding with its promoter [14]. As CaTFT7 interacts with CaHDZ27, we speculated that CaTFT7 can affect CaHDZ27 in the regulation of *CaCRK5* expression. To test this possibility, an electrophoretic mobility shift assay (EMSA) was carried out to analyze the effect of CaTFT7 on CaHDZ27 binding to the *CaCRK5* promoter. As shown previously, CaHDZ27 bound to the Cy5-labeled *CaCRK5* promoter. Furthermore, CaTFT7 did not bind to the *CaCRK5* promoter, but it dramatically enhanced the binding affinity of CaHDZ27 for the *CaCRK5* promoter when CaTFT7 proteins were added to the reaction mixture of CaHDZ27 (Fig. 5a).

Chromatin immunoprecipitation (ChIP) assays further confirmed that CaHDZ27 enrichment on the *CaCRK5* promoter increased upon *R. solanacearum* infection. This enrichment was significantly reduced in TRV:*CaTFT7* plants (Fig. 5b). Consistently, RT-qPCR analysis revealed reduced *CaCRK5* expression in *CaTFT7*-silenced plants following infection (Fig. S4). These findings suggested that CaTFT7 enhanced CaHDZ27 binding to the *CaCRK5* promoter, thereby upregulating *CaCRK5* expression.

In transient transactivation assays, the *proCaCRK5*:*GUS* (*CaCRK5* promoter fused with the β-glucuronidase gene), as the reporter, was transformed into *N. benthamiana* plants, and CaTFT7 and CaHDZ27 effector constructs were transiently expressed*.* The results indicated that GUS activity dramatically increased when either CaTFT7 or CaHDZ27 was transiently expressed relative to the control (empty vector). Moreover, higher GUS activity was detected when CaTFT7 and CaHDZ27 were coexpressed in *proCaCRK5*:*GUS* transgenic *N. benthamiana* leaves than CaHDZ27 alone (Fig. 5c). This supports the role of CaTFT7 in facilitating CaHDZ27-mediated transcriptional activation of *CaCRK5*.

In addition, we tested whether CaTFT7 affects the stability of CaHDZ27. Cell-free degradation assays indicated that accelerated degradation of CaHDZ27-GST in protein extracts from TRV:*CaTFT7* plants compared to TRV:*00* controls (Fig. 5d). Conversely, degradation was slower in extracts from *CaTFT7*-overexpressing plants (Fig. 5e and f). Furthermore, the degradation of CaHDZ27-GST was significantly inhibited when added to the MG132 (proteasome inhibitor), suggesting that the CaHDZ27 was indeed degraded via ubiquitin–proteasome system. These data suggest that CaTFT7 may function partially by preventing the proteasome degradation of CaHDZ27.

## Discussion

**Figure 6:**
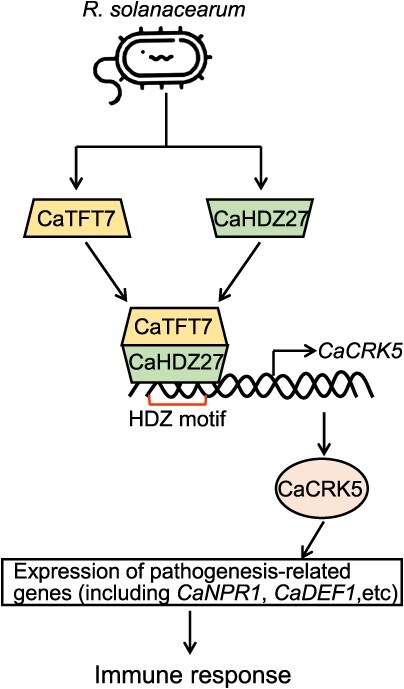
Working model of the roles of the CaTFT7-CaHDZ27 module in pepper immune response. *CaTFT7* and *CaHDZ27* were upregulated by *R. solanacearum* infection. CaTFT7 interacts with the TF CaHDZ27, which binds to the HDZ motif in the promoter region of *CaCRK5*. CaTFT7 promoted the stability of CaHDZ27 and enhanced its binding to the promoter of *CaCRK5*, thereby promoting the expression of pathogenesis-related genes and the immune response.

The 14-3-3 proteins are highly conserved, phosphorylated client-binding proteins [36]. In plants, 14-3-3 proteins exist in multiple isoforms and play a regulatory role in almost all aspects of cellular and physiological processes [37], suggesting that these 14-3-3 proteins have pleiotropic functions. In the present study, we identified a 14-3-3 protein, CaTFT7, that contributes to immunity against *R. solanacearum* in pepper by interacting with the TF CaHDZ27, which also plays a positive role in immunity against *R. solanacearum* [13]. When plants invade *R. solanacearum*, the transcript levels of *CaTFT7* and *CaHDZ27* increase. CaTFT7 increased the stability of CaHDZ27 and promoted its binding to the promoter of the target gene *CaCRK5* (Fig. 6). Thus, CaTFT7 positively regulates pepper immune response to *R. solanacearum* infection*.*

### CaTFT7 enhanced pepper resistance to *R. solanacearum.*

Increasing evidence has suggested that 14-3-3 proteins play a significant role in plant defense against pathogens [38], with some members serving as targets for pathogen effectors [18, 39]. In tomato, multiple 14-3-3 proteins, including SlTFT1, SlTFT4, SlTFT7, and SlTFT9, have been reported to participate in immunity [18, 29]. SlTFT4 and SlTFT7 are the targets of Type III effectors of *R. solanacearum* [*40**[.*

In the study, we characterized the 14-3-3 protein CaTFT7 in pepper defense response to *R. solanacearum*. Sequence analysis revealed that CaTFT7 contains a conserved 14-3-3 domain. Subcellular localization studies indicated that CaTFT7 is distributed in both the cytoplasm and nucleus. Upon *R. solanacearum* infection, *CaTFT7* transcript levels increased (Fig. 2a). Similarly, tomato SlTFT7 was found to accumulate in response to the invasion of *R. solanacearum* [41]*. CaTFT7*-silenced pepper plants presented attenuated resistance against *R. solanacearum* and decreased expression of the *CaPR1, CaNPR1*, and *CaDEF1* genes. Furthermore, transgenic *N. benthamiana* plants overexpressing *CaTFT7* presented increased resistance to *R. solanacearum.* Thus, our findings suggest that CaTFT7 positively regulates pepper resistance to *R. solanacearum*.

### CaTFT7 increased the transcriptional activity and stability of CaHDZ27

The 14-3-3 proteins exert their regulatory roles by interacting with target proteins, including TFs [33], even though they do not have DNA-binding ability [42]. The rice 14-3-3 protein OsGF14f positively modulates osmotic stress tolerance by interacting with OsbZIP23 to enhance its transcriptional activity. Similarly, our study revealed that CaTFT7 enhances CaHDZ27-mediated transcriptional regulation in pepper. Specifically, CaTFT7 promoted the binding of CaHDZ27 to the *CaCRK5* promoter (Fig. 5a), thus increasing the transcript level of *CaCRK5.* Furthermore, ChIP assays revealed that enrichment of the *CaCRK5* promoter was attenuated when CaTFT7 was silenced in pepper (Fig. 5b). Consistent with these findings, the induced expression of *CaCRK5* was compromised in *CaTFT7*-silenced pepper. These data confirmed that CaTFT7 interacted with CaHDZ27, increased its transcriptional activity, and played critical roles in the CaHDZ27-mediated pepper response to *R. solanacearum* infection.

Additionally, 14-3-3 proteins are known to regulate the stability of their interacting partners. For example, they destabilize cold-responsive C-repeat-binding factor (CBF) proteins during freezing stress [43]. Similarly, the rice protein GF14c enhances resistance to *Magnaporthe oryzae* by stabilizing TF OsSCL7 [23]. In this study, we found that CaTFT7 promoted the stability of CaHDZ27 (Fig. 5d and e), suggesting that CaTFT7 acted in pepper immunity partly by affecting the stability of CaHDZ27. Collectively, our data suggest that CaTFT7 participates in pepper resistance to *R. solanacearum* by increasing the transcriptional activity and promoting the stability of the TF CaHDZ27.

### Future perspectives on 14-3-3 proteins in plant immunity

The 14-3-3 proteins mediate the plant immune response by interacting with target proteins, but the underlying molecular mechanisms remain largely unexplored. In tomato, TFT7 positively regulates resistance protein-mediated programmed cell death. TFT7 interacts with MAPKKKα and MKK2 in the cytoplasm and acts as a scaffold protein to rapidly activate the MAP kinase cascade. In our study, we determined that CaTFT7 interacted with the TF CaHDZ27 to positively regulate the pepper immune response, suggesting that 14-3-3 proteins are involved in plant immunity through the diverse regulatory mechanisms.

Additionally, 14-3-3 proteins usually exert their functions by interacting with phosphorylated partners to regulate their functions [24]. Investigating whether CaHDZ27 undergoes phosphorylation will provide deeper insights into the regulatory role of CaTFT7 in plant immunity. Future research should focus on elucidating the phosphorylation dynamics of CaHDZ27 to better understand the molecular mechanisms underlying pepper immunity to *R. solanacearum*.

## Materials and methods

### Plant materials and growth conditions

Pepper (*Capsicum annuum* L., HN42) seeds were germinated in nutrition plates. After germination, plants were transplanted into pots and raised in the growth chamber at 28°C with 16 h/8 h (light/dark) photoperiod. *Nicotiana benthamiana* plants were grown in a growth chamber at 26°C under similar light/dark conditions.

### Pathogen inoculation

The *R. solanacearum* strain FJC100301 was cultured on nutrient agar at 28°C. Before inoculation, the *R. solanacearum* suspension was diluted to a concentration of 10^8^ CFU ml^−1^ using sterile water. In each pot, 1 ml of bacterial suspension was irrigated onto the wounded roots; sterile water was used as the control [44, 45]. The plants were subsequently cultured in a growth incubator at 70% relative humidity. Wilting symptoms were scored based on a scale ranging from 0 to 4. The disease index was calculated by averaging the disease scores of three independent experiments. For *R. solanacearum* infiltration in leaves, 50 μl of cell suspension (10^7^ CFU ml^−1^) was infiltrated into the leaves with a disposable syringe.

### cDNA synthesis and RT–qPCR

Total RNA was isolated from plant leaves using TRIzol reagent (Invitrogen, USA). cDNA was synthesized using the reverse transcription by a HiScript® Q RT SurperMix Kit (Vazyme, Nanjing, China) following the manufacturer’s instructions. qPCR was carried out via the SYBR Premix ExTaq II system (Takara). The relative expression levels were analyzed using the 2^−ΔΔCt^ method [46], with results presented as the means of three biological replicates. The gene-specific primers used for RT–qPCR are shown in Table S2.

### Virus-induced gene silencing

To suppress the expression of *CaTFT7* in pepper plants, VIGS was performed as described previously [47]. A specific 265-bp fragment from the coding region of *CaTFT7* was obtained to generate the pTRV2:*CaTFT7* construct, which was introduced into *Agrobacterium tumefaciens* strain GV3101. *Agrobacterium* cultures carrying pTRV1 were mixed with pTRV2:*CaTFT7*, pTRV2:*00*, or pTRV2:*CaPDS* (positive control) in a 1:1 ratio, and then infiltrated into the cotyledons of 3-week-old pepper seedlings (Fig. S5). The combination of pTRV1 and pTRV2:*CaPDS*, which causes an albino phenotype, was used as a positive control. After 3 weeks, the leaves at the top of the silenced pepper seedlings were inoculated with *R. solanacearum* as described above and then sampled for RT–qPCR.

### Electrophoretic mobility shift assay

The CaHDZ27-GST protein was obtained from a previous study [48]. CaTFT7–6 × His was purified with a BeaverBeads IDA-Nickel kit (Beaver Biosciences, China). The EMSA experiment was performed using CaHDZ27-GST, CaTFT7–6 × His, and the Cy5-labeled probes including the CaHDZ27 binding site (CAATTATTG) in the *CaCRK5* promoter. The reactions were analyzed through 6% (w/v) native polyacrylamide gels, and subsequently visualized by Odyssey CLx Imaging System (LI-COR Biosciences, USA).

### Subcellular localization

The coding sequence of CaTFT7 was subsequently cloned and inserted into a pEarleyGate101 vector to obtain a GFP fusion protein. The CaTFT7-GFP and NbH2B (histone H2B)-RFP (nucleus marker) plasmids were coexpressed in 4-week-old *N. benthamiana* leaves via the *Agrobacterium*-mediated method. At 48 hpi, GFP signals were observed via a laser scanning confocal microscope (TCS-SP8, Leica, Solms, Germany).

### Pull-down assay

CaHDZ27–6 × His and CaTFT7-GST were expressed in the *Escherichia coli* strain BL21 (DE3). The pull-down assay was conducted using anti-GST magnetic beads (Beaver Biosciences, China) as previously described [44]. Eluents were analyzed by immunoblotting with anti-His and anti-GST antibodies, with GST alone used as a negative control.

### Genetic transformation of *N. benthamiana*

The 35S:*CaTFT7-GFP* vector was introduced into *Agrobacterium* strain GV3101 for transformation into *N. benthamiana* plants via a leaf disc method [14]. Transgenic lines (T_0_) were confirmed by RT-PCR and propagated to obtain T_1_, T_2_, and T_3_ generations, which were further verified by PCR and western blot analysis using GFP antibodies.

### Bimolecular fluorescence complementation assay

The coding sequences of *CaTFT7* and *CaHDZ27* (excluding stop codons) were cloned into puc-SPYNE^GW^ and puc-SPYCE^GW^, respectively. Then, Constructs were coinfiltrated into *N. benthamiana* leaves, and YFP fluorescence was detected 48 hpi using a laser scanning confocal microscope.

### GUS activity assay

The 2000-bp promoter region of *CaCRK5* was inserted into pMDC163 to obtain the *pCaCRK5*:*GUS* construct. This was transformed into *N. benthamiana*. In the leaves of 4-week-old transgenic *N. benthamiana* plants, *35S*:*CaHDZ27-GFP* and *35S*:*CaTFT7-GFP* were transiently expressed. GUS activity was assayed via a Synergy H1 microplate reader (BioTek, USA) by monitoring the fluorescence of the produced 4-methylumbelliferone (4-MU) as described previously [49].

### Microscale thermophoresis assay

The MST assay was performed as described in a previous study [50]. CaHDZ27–6 × His and CaTFT7-GST were individually expressed in the *E. coli* strain BL21 (DE3) cells. Expression of the fusion protein was induced with IPTG (isopropyl β-D-1-thiogalactopyranoside) at 37°C for 5 h, and was dissolved in PBS (phosphate buffer saline). Then, CaHDZ27–6 × His was marked by fluorescence (Mo-L011, NanoTemper Technologies, Germany). CaTFT7-GST proteins were incubated with equal volumes of serial dilutions of purified CaHDZ27–6 × His protein at room temperature for 5 min. Then, the samples were loaded into glass capillaries (NanoTemper Technologies, Germany), and the essays were carried out via Monolith NT.115 An instrument (NanoTemper Technologies, Germany) with 50% IR laser power and an LED excitation source (λ =470 nm) was used. The data were analyzed via NanoTemper Analysis 1.2.20 software [51].

### Cell-free degradation assay

The cell-free degradation assay was performed as described previously [49]. Briefly, purified CaHDZ27-GST (500 ng) was incubated with crude protein extracts (50 μg total protein) from pepper or *N. benthamiana* for up to 60 min, with or without the proteasome inhibitor MG132 (50 μM). The mixture was collected at 0, 30, and 60 min separately, and detected by immunoblot with anti-GST antibody.

### Chromatin immunoprecipitation-qPCR

ChIP–qPCR assays were performed as described previously [49]. After crosslinking, immunoprecipitation was conducted with anti-GFP antibodies. DNA fragments were analyzed by qPCR using specific primers targeting CaHDZ27-binding regions in the *CaCRK5* promoter (Table S2). The enrichment value was normalized to that of the input sample.

## Supplementary Material

Web_Material_uhaf010

## Data Availability

The data that support the findings of this study are available in the manuscript and its online supplementary materials.
