# Supplementary material for: The 14-3-3 protein CaTFT7 interacts with transcription factor CaHDZ27 to positively regulate pepper immunity against Ralstonia solanacearum
Source: Hortic Res. 2025 Jan 14;12(4):uhaf010. doi: 10.1093/hr/uhaf010 (PMC11908829; doi:10.1093/hr/uhaf010)
Supplement: Web_Material_uhaf010 [file web_material_uhaf010.zip › Supplementary information.docx]

Table 1 Possible proteins that interact with CaHDZ27 in pepper.

| XP_016564341.1 | Luminal-binding protein 5 |
| --- | --- |
| XP_016547022.1 | Heat shock cognate 70 kDa protein |
| XP_016569248.1 | CaTFT7 |
| XP_016550223.1 | Catalase |
| XP_016540598.1 | GTP-binding nuclear protein Ran-3 |
| XP_016566289.1 | Metacaspase-4-like |
| XP_016579650.1 | Calmodulin-binding protein 60 B-like |
| XP_016565461.1 | U-box domain-containing protein 3 |
| XP_016542466.1 | Zinc finger CCCH domain-containing protein 11 |
| XP_016580213.1 | 26S protease regulatory subunit 8 homolog A |

Table S2 Information on all the primers used

| Genes | Assays | Primer sequences |
| --- | --- | --- |
| *CaTFT7* | ORF amplification | F ATGGTGCACTCTACTGTTTTTGTC |
|  |  | R TTAACTTAGAAAAGATTGAAGCATCCC |
|  | RT-qPCR | F GTGGAAGCAATGAAGGCGAT |
|  |  | R AGGAAGGAACAAGGTGCTCA |
|  |  | R GCCAACACATTCACCAGAGCATC |
|  | VIGS | F GTGGAAGCAATGAAGGCGAT |
|  |  | R AGGAAGGAACAAGGTGCTCA |
| *CaCRK5* | RT-PCR | F GGATTGCTCCGACTGCTTA  R TCCACTTTCTCCTCCTCAT |
|  | ChIP-qPCR | F TATAACCTTTTCTCCGAGTT |
|  |  | R TTAAGGGGCTATCGAGGC |
| *CaDEF1* | RT-qPCR | F GTGAGGAAGAAGTTTGAAAGAAAGTAC |
|  |  | R TGCACAGCACTATCATTGCATACAATTC |
| *CaNPR1* | RT-qPCR | F ACTTCTTCGCCGACGCCAAG |
|  |  | R GCCAACACATTCACCAGAGCATC |
| *CaPR1* | RT-qPCR | F GCCGTGAAGATGTGGGTCAATGA |
|  |  | R TGAGTTACGCCAGACTACCTGAGTA |
| *CaActin* | RT-qPCR | F AGGGATGGGTCAAAAGGATGC  R GAGACAACACCGCCTGAATAGC |
| *NbEF-1α* | RT-qPCR | F TGCTGCTGTAACAAGATGGATGC |
|  |  | R GAGATGGGGACAAAGGGGATT |


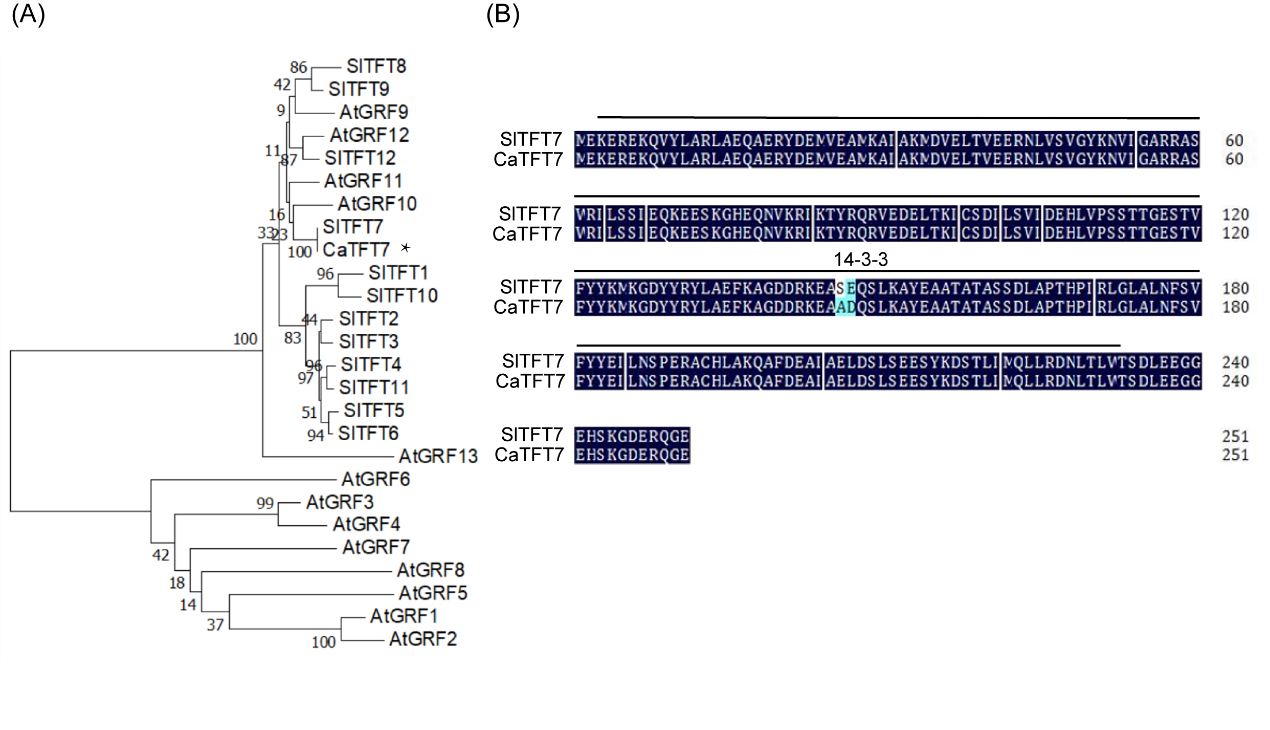


Figure S1 (A) Phylogenetic tree of CaTFT7 and 14-3-3 members from tomato and *Arabidopsis* via the neighbor-joining method in MEGA 7.0 with 1,000 bootstrap replicates. The numbers next to each node indicate confidence percentages. The asterisk indicates CaTFT7. (B) Amino acid comparison of pepper CaTFT7 and tomato SlTFT7. The 14-3-3 domain is indicated with a solid line.


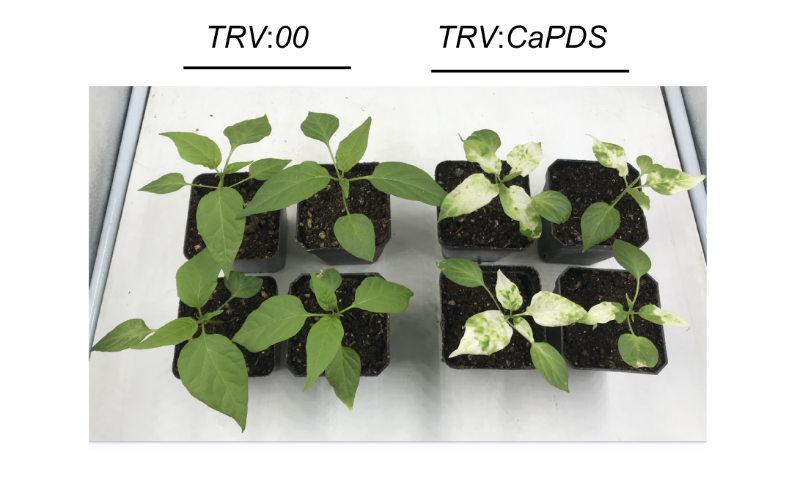


Figure S2 Photobleaching phenotype of *CaPDS-*silenced pepper plants. Images of five-week-old TRV:*CaPDS* and TRV:*00* pepper plants were taken.


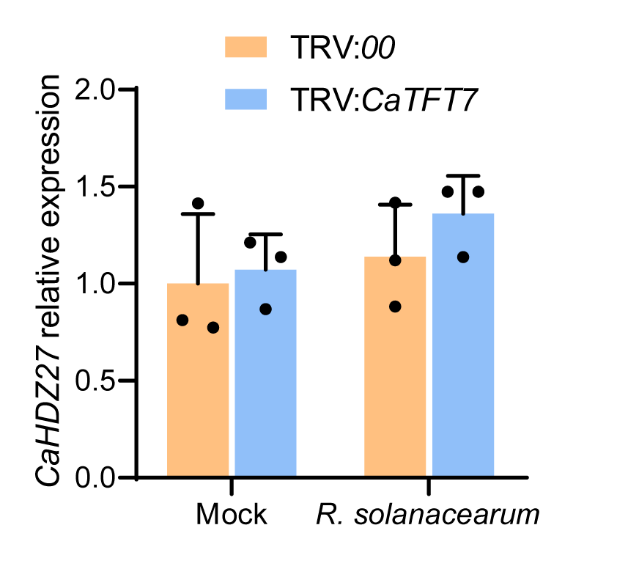


Figure S3 The transcript level of *CaHDZ27* in *CaTFT7* silenced pepper plants by RT‒qPCR. The data are presented as the means ± SDs of three biological replicates.


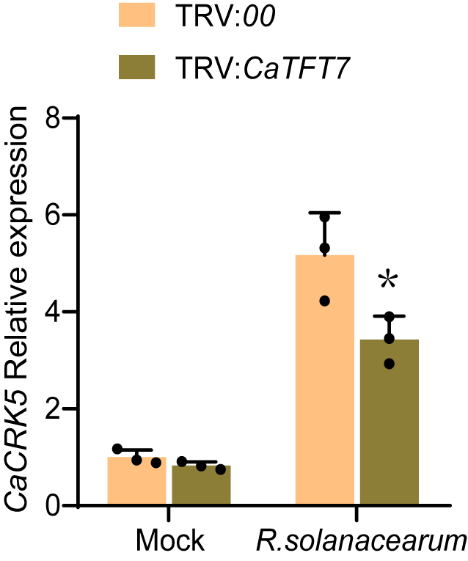


Figure S4 The transcript level of *CaCRK5* in *CaTFT7* silenced pepper plants by RT‒qPCR. The data are presented as the means ± SDs of three biological replicates. * *P* < 0.05 (Student’s t test).


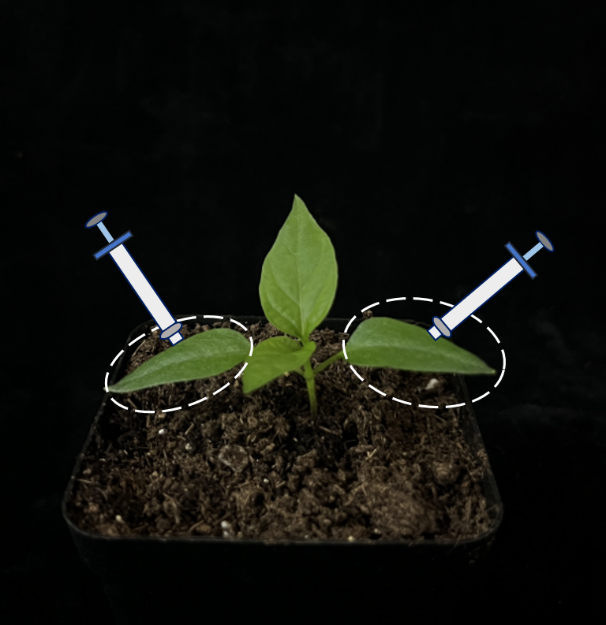


Figure S5 The diagram illustrating that two pepper leaves are infiltrated by *Agrobacterium* in VIGS essay.
